# Aural Polyp or Temporal Bone Carcinoma: Lesson to Learn

**DOI:** 10.7759/cureus.13629

**Published:** 2021-03-01

**Authors:** Khairil Afif Mahmud, Zara Nasseri, Shahizon Azura Mohamed Mukari, Fuad Ismail, Asma Abdullah

**Affiliations:** 1 Department of Otorhinolaryngology - Head and Neck Surgery, Universiti Kebangsaan Malaysia Medical Centre, Kuala Lumpur, MYS; 2 Department of Radiology, Universiti Kebangsaan Malaysia Medical Centre, Kuala Lumpur, MYS; 3 Department of Oncology and Radiotherapy, Universiti Kebangsaan Malaysia Medical Centre, Kuala Lumpur, MYS

**Keywords:** aural polyp, temporal bone carcinoma

## Abstract

Temporal bone carcinoma is a rare malignant tumor of the head and neck region. Its clinical presentations can mimic benign ear diseases, leading to inaccurate diagnosis and substandard management. We present the case of a 53-year-old female with a three-month history of progressive right otalgia, otorrhea, and hearing loss. Otoscopic examination revealed a mass occupying the right external auditory canal. However, the lesion was presumed to be an aural polyp by several clinicians previously. Multiple courses of oral antibiotics had been prescribed before she was referred to our clinic for the non-resolving aural polyp. Imaging studies showed an external auditory canal soft tissue mass with extradural and parotid extension. The mass was biopsied, and the result was reported as squamous cell carcinoma of the temporal bone. The patient was advised for a total temporal bone resection and parotidectomy; however, she declined the surgical intervention. Within a month, the tumor had metastasized to her lung, liver, and vertebral bodies. She was referred to the Oncology team for palliative chemo-radiotherapy. Temporal bone malignancy must be considered as a differential diagnosis in a middle-aged or elderly patient with a non-resolving aural polyp without a chronic discharging ear. Imaging studies and histopathological evaluation should be prompted to ascertain the diagnosis. Repeated course of oral antibiotic will delay treatment and subsequently may lead to poor prognosis.

## Introduction

Temporal bone carcinoma is an exceedingly rare tumor of the head and neck region (less than 0.2%), and it carries a good survival rate if detected early during the disease course [1]. However, the diagnosis can be missed as the clinical presentations can simulate benign ear diseases [2]. A delay in diagnosing temporal bone carcinoma may allow the tumor to progress and leads to poor prognosis.

## Case presentation

**Figure 1:**
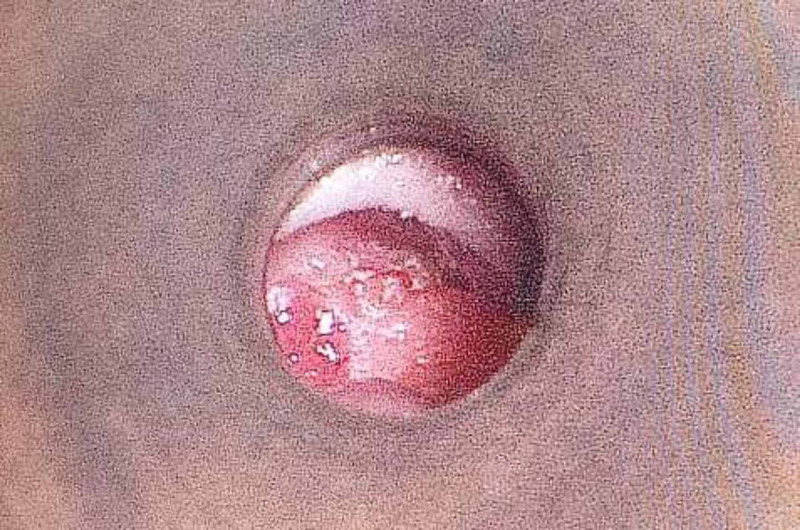
Microscopic view of the right external auditory canal.

**Figure 2:**
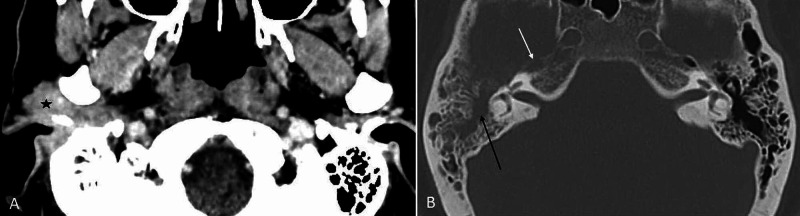
CT of the brain. (A) Contrast-enhanced CT demonstrating an enhancing lesion at the right external auditory canal, pre-auricular region (black star). The ipsilateral mastoid air cells are not aerated filled with soft tissue density, while preserving the bony septation. (B) CT in the bone setting showing the mass occupying the middle ear cavity with the destruction of the incudomalleal complex (black arrow). The anterior cortical margin of the right petrous apex is destructed (white arrow). CT, computed tomography

A 53-year-old female of Malay ethnicity presented with progressive otalgia, associated with blood-stained otorrhea and reduced hearing. Three months prior, she had visited multiple general practitioners and was treated for an aural polyp. She had received repeated courses of oral antibiotics before she was referred to our clinic for the non-resolving aural polyp. She had no history of a chronic discharging ear. Otoscopic examination revealed a pink-colored mass occupying the entire external auditory canal and obscuring the view of the tympanic membrane (Figure 1). There was no facial palsy or other cranial nerve palsies. A temporal bone high-resolution computed tomography (HRCT) was performed and it showed a heterogenous enhancing soft tissue lesion in the right ear canal, extending into the right mastoid cavity and the middle ear (Figure 2).

**Figure 3:**
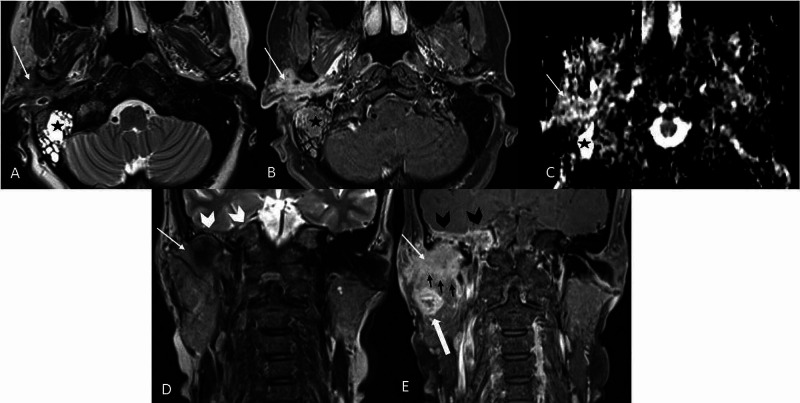
MRI of the base of skull and neck in (A) axial T2-weighted image, (B) axial T1 post-contrast with fat suppression, (C) ADC map, (D) coronal T2-weighted image, and (E) coronal T1 post-contrast with fat suppression. The lesion is hypointense on T2, enhances homogeneously, and shows mix low and high signals on ADC (white arrow). Loss of the hypointense bony cortex of the petrous apex and the floor of the middle cranial fossa (white arrowhead) results in extension of the lesion/inflammation to the right petrous apex; note the involvement of the dural reflection at the floor of the right middle cranial fossa (black arrowhead). The lesion infiltrates the parotid gland inferiorly (black arrow). Intraparotid necrotic node is present (block white arrow). The lesion demonstrates an infiltrative pattern, lacking a significant mass effect. Retention fluid occupies the right mastoid air cells (black star). MRI, magnetic resonance imaging; ADC, apparent diffusion coefficient

She underwent examination under general anesthesia and biopsy of the ear canal mass. Histopathological examination was reported as well-differentiated squamous cell carcinoma. She then underwent magnetic resonance imaging (MRI) that demonstrated a cortical breach of the middle cranial floor with extradural tumor extension. Inferiorly, the lesion involved the superior aspect of the ipsilateral parotid gland (Figure 3). She was counseled for right total temporal bone resection and parotidectomy with ipsilateral selective neck dissection. Unfortunately, she refused surgical intervention at that time. A subsequent scan one month later revealed local disease progression with extensive extradural involvement, extending into the inferior cranial fossa. The lesion had also metastasized to the lungs, liver, and vertebral bodies. Thus, she was referred to the oncology team for palliative chemo-radiotherapy.

## Discussion

The incidence rate of temporal bone carcinoma ranges from one to six cases per million a year. Thus, the awareness about this disease is still lacking. Acharya et al. observed that the incidence of temporal bone carcinoma is the highest among the age groups of 60-70 and 75-79 years [1]. Other significant risk factors include prior radiation, recurrent ear infection, and cholesteatoma. Nevertheless, none of these features presents in the current case except for the patient’s age. Almost half of the patients with temporal bone malignancy present with otorrhea, otalgia, and hearing loss, although these symptoms are also seen in non-malignant conditions [2]. Clinically, temporal bone carcinoma can manifest as ear canal mass; however, it is only detected in 12.7% of the cases according to Gidley et al. [2,3].

A malignant aural polyp is a possible diagnosis for patients presenting with blood-stained otorrhea, but this is unusual. It can be a primary malignancy or a result of metastasis, mainly from parotid or nasopharyngeal carcinoma. Tissue sampling should be performed for all aural polyps that do not respond to medical treatment or without an identifiable origin [4]. In real practice, otoscopic findings sometimes can be difficult to visualize deeper structures, and without adequate visualization, tissue biopsy taken peripherally may not represent the correct pathology. We postulate that the first biopsy taken initially was superficial and not representative of the mass. Imaging should be done early as it can navigate diagnosis and surgical planning [2,5]. HRCT can demonstrate temporal bone erosion which suggests aggressive inflammation or malignancy. MRI complements by providing a detailed evaluation of the soft tissue, where the extradural extension was not previously appreciated in the HRCT [6].

The mainstay treatment of temporal bone squamous cell carcinoma is surgery except in cases where the patient is surgically unfit, the tumor is irresectable, or the patient develops distant metastasis. The surgery can be either lateral, subtotal, or total temporal bone resection, depending on the extent of the disease. Additional superficial parotidectomy with level II and III neck dissection is recommended when there is evidence of cervical lymph node metastasis [7]. Moody et al. reported almost 100% two-year survival rate in early-stage tumors (T1, T2), whereas the survival rate decreased by more than half in advanced stages (T3, T4) [8].

## Conclusions

Early detection of temporal bone carcinoma is crucial as the prognosis is excellent in the early stage. It should be suspected in all middle-aged or elderly patients with a non-resolving aural polyp, especially those without preceding ear infections. HRCT of the temporal bone should be requested urgently followed by urgent biopsy of the mass. Multiple oral antibiotic treatments caused a delay in the treatment, allowing the tumor to progress into advanced stage, which has a low survival rate.
